# Modulation of T Cell Function by Combination of Epitope Specific and Low Dose Anticytokine Therapy Controls Autoimmune Arthritis

**DOI:** 10.1371/journal.pone.0000087

**Published:** 2006-12-20

**Authors:** Sarah T.A. Roord, Evelien Zonneveld-Huijssoon, Tho Le, Gisella Puga Yung, Eva Koffeman, Arash Ronaghy, Negar Ghahramani, Paola Lanza, Rosario Billetta, Berent J. Prakken, Salvatore Albani

**Affiliations:** 1 Department of Medicine, University of California San Diego La Jolla, California, United States of America; 2 Department of Pediatrics, University of California San Diego La Jolla, California, United States of America; 3 Department of Pediatric Immunology, University Medical Center Utrecht, Wilhelmina Children's Hospital Utrecht, The Netherlands; 4 Androclus Therapeutics San Diego, California, United States of America; 5 Immunology Advanced Center On Preclinical Immunogenomics project, EUREKA Institute for Translational Medicine Siracusa, Italy; New York University School of Medicine, United States of America

## Abstract

Innate and adaptive immunity contribute to the pathogenesis of autoimmune arthritis by generating and maintaining inflammation, which leads to tissue damage. Current biological therapies target innate immunity, eminently by interfering with single pro-inflammatory cytokine pathways. This approach has shown excellent efficacy in a good proportion of patients with Rheumatoid Arthritis (RA), but is limited by cost and side effects. Adaptive immunity, particularly T cells with a regulatory function, plays a fundamental role in controlling inflammation in physiologic conditions. A growing body of evidence suggests that modulation of T cell function is impaired in autoimmunity. Restoration of such function could be of significant therapeutic value. We have recently demonstrated that epitope-specific therapy can restore modulation of T cell function in RA patients. Here, we tested the hypothesis that a combination of anti-cytokine and epitope-specific immunotherapy may facilitate the control of autoimmune inflammation by generating active T cell regulation. This novel combination of mucosal tolerization to a pathogenic T cell epitope and single low dose anti-TNFα was as therapeutically effective as full dose anti-TNFα treatment. Analysis of the underlying immunological mechanisms showed induction of T cell immune deviation.

## Introduction

Much progress has recently been achieved on our knowledge of the immunological and molecular mechanisms, which lead to amplification, and perpetuation of autoimmune inflammation. This progress has been translated into a generation of biologic therapeutic agents that target pro-inflammatory cytokines, with the aim of interfering with their mechanism of action. This approach is destined to progressively complement and, in some cases, replace currently used immunosuppressive and anti-inflammatory therapies. Despite their success[1], [2], current anti-cytokine approaches remain hampered with limitations associated eminently with generalized immunosuppression and subsequent increased occurrence of malignancies and infectious diseases, in particular tuberculosis[3]–[6].

Conceptually, therapeutic intervention focused on modulation of T cell function leads to the promise of higher specificity and lower toxicity[7]–[16]. This objective has for long remained a challenge in humans, particularly due to the difficulty of identifying means of intervention that could affect T cell function in a specific fashion.

In a Phase I/IIa trial, we have recently described immunological effects of epitope specific immunotherapy in a group of patients with rheumatoid arthritis. The epitope employed was derived from the heat shock protein (HSP) dnaJ. We have proposed a central role for HSP-specific T cell responses in the physiologic mechanisms of modulation of inflammation[17]–[20]. We have also suggested impairment of such modulation as one of the mechanisms of amplification of autoimmune inflammation[21]–[24]. Our treatment sought to restore such control by inducing mucosal tolerization to a peptide with a potential pathogenic, not necessarily etiologic role[25]. Immunological effects of the treatment consisted of immunodeviation from pro-inflammatory to tolerogenic type T cell responses to the peptide employed in the treatment. Restoration of regulatory T cell activity was also observed.

Effects of anti cytokine therapy on T cell function, both effector and regulatory, have been suggested[13], [26]–[28]. These interactions are relevant for many different reasons, including ultimately the design of an optimal biologic therapy based on the combination of anti-cytokine and T cell epitope specific approaches.

The work presented here lays the foundation for this strategy by exploring clinical and immunological effects of the combination of epitope specific T cell and anti-cytokine therapy. We employed for this purpose Adjuvant Arthritis (AA). This is an experimental form of arthritis that is T cell dependent and can be passively transferred by a T cell clone that is specific for the 180-188 amino acid sequence of mycobacterial HSP60[29], [30]. In previous studies we showed that nasal administration of a 15-mer peptide (176-190) encompassing this arthritogenic epitope leads to T cell tolerance[31] and can prevent AA. Treatment with nasal administration of peptide 180-188 after the induction of AA is mildly effective. Here, we compared immunological and clinical effects of different dose regimens, namely full dose anti-TNFα, which is known to be effective[32], mucosal tolerization to the peptide alone, anti-TNFα at one third of the effective dose, and the combination of low dose anti-TNFα and epitope specific therapy.

We found that the combination of low dose anti-TNFα associated with mucosal tolerization to the arthritogenic T cell epitope led to a significant reduction of arthritis clinically as well as histologically, to a degree entirely comparable with what was achieved with full dose anti-TNFα. Interestingly, treatment regimens differed for their influence on immune responses. Indeed, combination therapy induced T cells with a regulatory phenotype, consisting of CD4+CD25+ cells producing IL-10 and expressing FOXP3. Combination treatment also induced immune deviation in CD4+CD25− cells, which were found producing IL-10, as well as IL-4. Such changes were not present in the full dose anti-TNFα therapy group.

Our data provide a compelling rationale for testing the combination of anti-cytokine and epitope specific immunotherapy in human autoimmune disease.

## Methods

### Animals

Male inbred Lewis rats (RT1B) were obtained from Harlan (Indianapolis). Rats were 6–9 weeks old at the start of each experiment.

### Antigens and adjuvants

Heat killed *Mycobacterium tuberculosis* (Mt, strain H37Ra) was obtained from Difco (Detroit, MI). Incomplete Freund's Adjuvant (IFA; Difco, Detroit, MI) was used as adjuvant. The peptide used in this study was prepared in large quantities by standard solid phase Fmoc chemistry. It was obtained as COOH terminal amide and was analyzed and purified by reverse-phase HPLC. The following peptide was used: *Mycobacterium tuberculosis* HSP60 180-188, containing *Mycobacterium tuberculosis* HSP60 sequence 180-188 (TFGLQLELT). 180-188 is recognized by the arthritogenic T cell clone A2b and is a dominant T cell epitope found after Adjuvant Arthritis (AA) and after immunization with mycobacterial HSP60.

### Induction and Clinical Assessment of Experimental Arthritis

Rats were lightly anesthetized using isoflurane and AA was induced by a single intradermal (i.d.) injection in the base of the tail with 0.1 mg *Mycobacterium tuberculosis* (Mt) suspended in 100 µl of IFA (Complete Freund's Adjuvant; CFA). Rats were examined daily for clinical signs of arthritis in a blinded set-up. Severity of arthritis was assessed by scoring each paw from zero to four based on degree of swelling, erythema and deformation of the joints. Thus the maximum score was 16. On day 23 after the induction of arthritis the rats were sacrificed by CO2 inhalation, after which mandibular lymphnodes (MLN), Inguinal Lymphnodes (ILN), spleen and hind limb joints were collected.

### Immunotherapy Protocols

Rats were lightly anesthetized using metofane for all nasal treatments or isoflurane for all subcutaneous treatments. Etanercept (Enbrel®, Wyeth) was administered subcutaneously (s.c.) at a concentration of 0.3 mg/kg per rat dissolved in 100 µl PBS using a 25-gauge needle. This was done on day 9 after the induction of arthritis with Mt. Some rats in control groups received additional Etanercept on day 11 and 13. 100 µg of peptide dissolved in PBS was administered nasally in a total volume of 10 µl (5 µl per nostril, peptide concentration 10 mg/ml) using a micropipette. This was done on day 10, 13, 16, 19 after arthritis induction with Mt.

### Adoptive transfer

MLN, ILN and spleen of 2–3 rats per group after combination treatment with Etanercept and 180-188 were harvested on day 23 after the induction of arthritis. Cells were cultured in vitro with 2.5 µg/ml conA for 48 hours. Subsequently, 13×10^6^ MLN, 11×10^6^ ILN and 11×10^6^ spleen cells were injected i.v. into the tail vein of rats one week after induction of arthritis with Mt. Rats were subsequently examined daily for clinical signs of arthritis in a blinded set-up as described previously.

### Histological assessment of hind limb joints

Hind limb joints were collected on day 23 after the induction of arthritis, after the rats were sacrificed by CO2 inhalation. Formalin-fixed tissues were decalcified, and glass slides stained with H&E and Safranin O (for cartilage) were prepared and evaluated by standard methodology (Comparative Biosciences, Inc.). The pathologist examined all of the submitted tissue sections in a blinded fashion by light microscopy and scored for inflammation of the synovium, pannus formation, cartilage damage, inflammation of the bone marrow and periostal proliferation. Each of these parameters was scored for 10 days, severity from 0 (normal) to 4 (severe). A cumulative score was given based upon the sum of all of the parameters measured.

### Intracellular Cytokine Staining

MLN cells were cultured for 72 hours with medium alone or antigen. During the last 4 hours of culture 1 M monensin (GolgiStop®, Pharmingen, San Diego, CA) was added. Viable cells were harvested and washed with FACS blocking buffer (PBS with 10% FBS) and 0.03% 1 M sodium azide) and subsequently stained for 30 minutes on ice in 100 µl of blocking buffer with the following conjugated monoclonal antibodies for extracellular antigens: PE, FITC or CY-conjugated anti-rat CD4 (clone OX-35, mouse IgG2a), FITC-conjugated anti-rat CD25 (clone OX-39, mouse IgG1) (BD Pharmingen, San Diego CA). The cells were washed twice in staining buffer (PBS containing 3% FBS and 0.03% 1 M sodium azide) and resuspended in 100 µl fixation buffer (Cytofix/Cytoperm®, BD Pharmingen, San Diego, CA) for 20 minutes on ice. The cells were washed twice in permeabilization buffer (Perm/Wash®, Pharmingen, San Diego CA) and resuspended in 100 µl permeabilization buffer and stained with the following conjugated monoclonal antibodies: PE-conjugated anti-rat IL-4 (clone OX-81, mouse IgG1), PE-conjugated anti-rat IL-10 (clone A5-4, mouse IgG2b), PE-conjugated anti-rat TNFα (clone TN3.-19.12, hamster IgG) and PE-conjugated anti-mouse CTLA-4 (anti CD152) (clone UC10-4F10-11, armenian hamster IgG, group 1κ) (all antibodies from Pharmingen, San Diego, CA). The appropriate isotype controls were used. Finally, the cells were washed twice, resuspended in staining buffer, and transferred to FACS tubes for analysis. Stained cells were analyzed on a FACStar Plus cytometer (Becton and Dickinson). At least 5.000 events were acquired from each sample and subsequently analyzed with Lysis II software.

### Sorting of CD4+CD25+ and CD4+CD25− after magnetic bead separation

MLN were incubated for 15 hours with medium or antigen. Viable cells were harvested and first the cell suspensions were depleted of monocytes, phagocytes, NK cells and B cells by magnetic bead separation using the CELLection®Biotin Binder kit (Dynal A.S. Oslo, Norway). In brief, cells were incubated with the following monoclonal antibodies: biotin mouse anti rat mononuclear phagocyte, (C17, Pharmingen), biotin mouse anti rat CD161a (10/78, Pharmingen) and biotin mouse anti rat CD45RA (OX-33, Caltag Laboratories). Positive selection was performed using streptavidin coated magnetic Dynabeads using the Dynal Magnetic Particle Concentrator.

The thus obtained cells were washed in FACS blocking buffer and stained extracellularly with anti rat CD4 and anti rat CD25. Subsequently, cells were sorted by FACS (FACS Vantage, Beckton Dickinson San Jose, CA) into CD4+CD25+ and CD4+CD25− cells.

### Real Time Quantitative PCR (Taqman)

MLN were incubated for 15 hours with medium or antigen. Cells were sorted into CD4+CD25+ cells and CD4+CD25− cells as described above, resuspended in Lysis buffer (Qiagen,Valencia, CA) and frozen at −80°C until analysis. mRNA was extracted from sorted cells by using RNeasy Mini Kit (Qiagen). mRNA was then reverse-transcribed into cDNA with an oligo dT primer (Oligo(dT)12-18, Invitrogen). Subsequently, single stranded cDNA was amplified with the cytokine specific forward and reverse primer sets for GAPDH (housekeeping gene), IL-10, TNFα and FOXP3. mRNA levels were determined by Real Time Quantitative PCR on an ABI PRISM® 7000 thermal cycler (Perkin Elmer). The following combinations of primers and probes were used: IL-10 Forward 5′GCC TGG CTC AGC ACT GCT AT 3′, IL-10 Reverse 5′CGG ATG GAA TGG CCT TTG 3′, IL-10 Probe-FAM 5′ TTG CCT GCT CTT ACT GGC TGG AGT GAA 3′. TNFα Forward 5′ACA AGG CTG CCC CGA CTA C 3′, TNFα Reverse 5′TCC TGG TAT GAA ATG GCA AAC C 3′, TNFα Probe-JOE 5′TGC TCC TCA CCC ACA CCG TCA GC 3′. FOXP3 Forward 5′CCA TTG GTT CAC ACG CAT GT 3′, FOXP3 Reverse 5′TGG CGG ATG GCA TTC TTC 3′, FOXP3 Probe-JOE 5′CGC CTA CTT CAG AAA CCA CCC 3′. GAPDH Forward 5′TGA CTC TAC CCA CGG CAA GTT 3′, GAPDH Reverse 5′TTC CCG TTG ATG ACC AGC TT 3′, GAPDH Probe-FAM 5′ACG GCA CAG TCA AGG CTG AGA ATG G 3′.

To quantify the amount of mRNA for the different target genes the standard curve method was used[33].The relative amounts of target gene and GAPDH were quantified by a linear extrapolation of the Ct values using the equation to the line obtained from the standard curve of the respective target genes. Data were normalized for target gene expression, which was obtained by dividing the relative quantity of target gene for each sample divided by the relative quantity of GAPDH for the same sample. The final read outs are expressed as induction index (arbitrary units) defined as stimulated subtracted by reference condition, *i.e.* only media culture.

### Statistical analysis

A two tailed, paired t-test was performed to compare clinical scores on day 23 and to compare Area under the arthritis score curve. Kolmogorov-Smirnov Statistics were applied for statistical analysis of FACS histograms.

## Results

### Combination of epitope specific therapy and a single low dose of Etanercept (Enbrel®) has clinical efficacy comparable to full dose Etanercept in controlling Adjuvant Arthritis

Lewis rats were immunized with 100 µg Mt to induce AA and randomly divided into 5 treatment groups: i) no treatment; ii) three doses of Etanercept s.c., equivalent in this model to a full course of Etanercept treatment; iii) one dose of Etanercept s.c.; iv) four nasal administrations of HSP60 peptide 180-188 v) combination of one dose of Etanercept s.c. followed by four nasal treatments with HSP60 peptide 180-188. Three independent experiments were performed, with 6 rats per treatment group. The lowest effective dose of Etanercept was determined in preliminary experiments (not shown). Two different parameters were employed to measure clinical outcomes, in order to ascertain full evaluation of the effects of the various treatment regimens: i) mean arthritis scores on day 23 (the day of maximum arthritis severity); ii) area under the curve of the corresponding arthritis score curves, thus taking into consideration the whole time course of the treatment.

A significant reduction of AA mean arthritis scores on day 23 (p = 0.0004) was achieved with epitope specific and low single dose Etanercept combination treatment as well as with a full course of Etanercept therapy (p = 0.004) compared to no treatment. Similarly, when assessing the areas under the curve (AUC) of the corresponding arthritis score curves, a significant decrease of AA was seen after epitope specific and low single dose Etanercept combination treatment (p = 0.02 vs. no treatment). Comparable disease control was achieved in the full dose Etanercept treatment group (p = 0.03 vs. no treatment).

**Figure 1:**
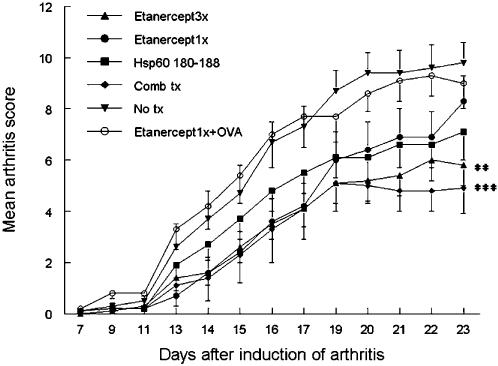
Combination therapy of Etanercept with mycobacterial heat shock protein 60 (HSP60) 180-188 led to significant reduction of Adjuvant Arthritis (AA). Arthritis was induced on day 0 with Complete Freund's Adjuvant (CFA). On day 9, rats were randomly divided into five treatment groups: three doses of Etanercept s.c. on day 9, 11, 13 (equivalent to a full course of Etanercept treatment); one dose of Etanercept on day 9; four doses of mycobacterial HSP60 peptide 180-188 on day 10, 13, 16, 19; combination treatment of one dose of Etanercept s.c. on day 9 followed by 180-188 nasally on day 10, 13, 16, 19; or no treatment (PBS). Arthritis scores were assessed every other day from day 8 onward. N = 15–18 rats per treatment group. Shown are mean arthritis scores.

One dose of Etanercept alone on day 9 was able to suppress arthritis only temporarily; however, as expected, after day 17 the arthritis revived (Day 23 p = 0.3, AUC p = 0.1 vs. no treatment). Treatment with HSP60 peptide 180-188 alone showed a trend towards reduction of arthritis, without achievement of statistically significant differences (Day 23 p = 0.07, AUC p = 0.26 vs. no treatment). Combination of treatment with an irrelevant peptide, derived from Ovalbumin, and low dose Etanercept lacked efficacy in suppressing arthritis, thus confirming the epitope specificity of the treatment (Figure 1 and Table 1).

**Table 1:**
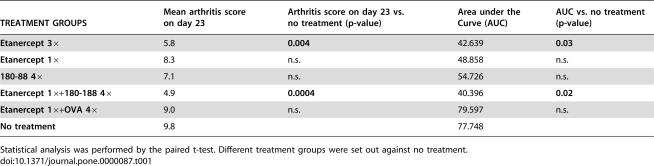
Combination treatment as well as a full course Etanercept treatment led to significant reduction of arthritis on day 23, when maximum score of disease is reached, as well as a significant reduction of the area under the arthritis score curve (AUC), representative of the whole treatment period.

TREATMENT GROUPS	Mean arthritis score on day 23	Arthritis score on day 23 vs. no treatment (p-value)	Area under the Curve (AUC)	AUC vs. no treatment (p-value)
**Etanercept 3×**	5.8	**0.004**	42.639	**0.03**
**Etanercept 1×**	8.3	n.s.	48.858	n.s.
**180-88 4×**	7.1	n.s.	54.726	n.s.
**Etanercept 1×+180-188 4×**	4.9	**0.0004**	40.396	**0.02**
**Etanercept 1×+OVA 4×**	9.0	n.s.	79.597	n.s.
**No treatment**	9.8		77.748	

Statistical analysis was performed by the paired t-test. Different treatment groups were set out against no treatment.

Hence, regardless of the outcome parameter employed, combination of epitope specific and low single dose Etanercept therapy enabled complete clinical control of the arthritic process to a degree statistically comparable with full dose Etanercept, a therapeutic regimen known to fully control AA.

### Epitope specific and low single dose Etanercept combination therapy leads to a decrease of damage in the hind limb joints

Next we investigated if clinical control of AA with combination therapy was matched in the same treatment groups by a corresponding decrease in joint destruction by the arthritic process. Hind limb joints were collected on day 23 after the induction of arthritis and scored for severity of inflammation in the synovium, pannus formation, cartilage damage, inflammation of the bone marrow and periostal proliferation, with a maximum total score of 20.

**Figure 2:**
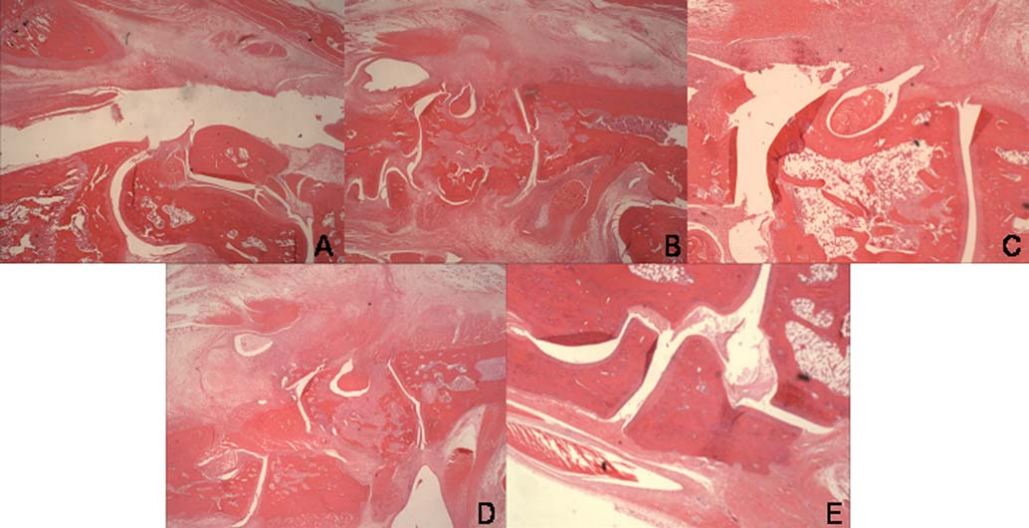
Combination therapy as well as a full course of Etanercept treatment led to reduction of histological damage in the ankle joints. Joints were harvested on day 23 after the induction of arthritis. Formalin-fixed tissues were decalcified, and glass slides stained with H&E were prepared. Submitted tissue sections were examined by light microscopy and scored for severity of inflammation of the synovium, pannus formation, cartilage damage, bone marrow inflammation and periostal proliferation, with a maximum score of 4 per parameter. N = 3–4 per treatment group. H&E staining is shown of one rat per treatment, representative for the whole treatment group. A: Combination therapy; B: Etanercept 1×; C: Peptide Mt. 180-188 4× monotherapy; D: No treatment; E: Etanercept 3×.

Epitope specific and single low dose Etanercept combination therapy led to a significant improvement of the histological score in the joints (p = 0.014 vs. untreated). Similarly, full course of anti-TNFα therapy led to a significant decrease of histological damage (p = 0.001 vs. no treatment). Single dose of Etanercept did not lead to significant improvement (p = 0.214) (Figure 2).

### Epitope specific and single low dose Etanercept combination therapy induces immune deviation of CD4+ T cells

**Figure 3:**
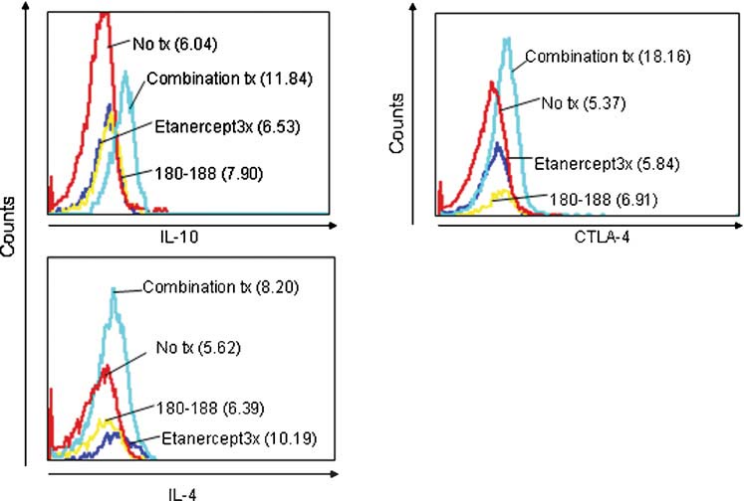
The combination therapy of Etanercept and HSP60 180-188 led to an antigen specific increase of IL-10 and IL-4 production and up regulation of CTLA-4 expression in CD4+ T cells in draining Mandibular Lymphnodes (MLN). MLN were harvested on day 23 after the induction of arthritis. Cells were cultured for 72 hours with medium or antigen. Intracellular production of IL-4, IL-10, and expression of CTLA-4 were measured by FACS. Depicted are Mean Fluorescence Indexes (MFI) of MLN cells cultured with mycobacterial HSP60 peptide 180-188, of cells gated on CD4. Results are representative of one experiment.

We then analyzed the immune mechanism responsible for the clinical effects of the treatments tested. We focused in this part of our analysis on defining qualitatively CD4+ mediated T cell responses to the inciting antigen. The rationale behind this strategy was to identify qualitative changes in cytokine responses induced by the treatment. To this end, we measured cytokine production and surface marker expression of CD4+ T cells present in the Mandibular Lymphnodes (MLN), the draining site of the nasal mucosa where T cell immune deviation may be induced. MLN were isolated on day 23 after arthritis induction and cultured with HSP60 peptide 180-188 or media. It has to be noted that HSP60 peptide 180-188 is the major immune dominant epitope following induction of AA (due to the presence of mycobacterial HSP65 in CFA) and thus can act as an important surrogate parameter for (immune) therapy in AA[34], [35]. After 72 hours viable cells were harvested and stained for surface markers and intracellular cytokines and analyzed by FACS. The results showed differences between treatment groups in the immune mechanisms underlying sometimes comparable clinical efficacy. In fact, only the epitope specific/low dose Etanercept combination treatment group showed clear indication of T cell immune deviation, as indicated by the significant difference compared to the untreated group as well as the Etanercept groups in the increased production of IL-10 regulatory cytokine (MFI 11.84, isotype control 7.69, p<0.001). Expression of CTLA-4, a marker of T cells with regulatory function, also significantly increased when compared both to untreated and Etanercept groups (MFI 18.16, isotype control 7.10, p<0.001). An increase in IL-4 production was seen after the combination treatment as well as after the full course of Etanercept therapy (MFI 8.20 and 10.19, isotype control 6.96, p<0.001) (Figure 3).

These data are, in our opinion, intriguing as they show differences in underlying immunological mechanism between two equally clinically effective treatments. Indeed, the marked increases in IL-10 production and CTLA-4 expression following combination therapy were both strongly suggestive of restored modulation of T cell function.

### Enhancement of CD4+CD25+ regulatory T cell (Treg) function by epitope specific/low dose anticytokine combination therapy

In this part of the project, we addressed the questions on: i) whether certain aspects of regulatory T cell (Treg) function were affected by the combination therapy; ii) whether such induction would affect immune deviation in effector T cells; iii) whether differences in these parameters between full dose Etanercept and epitope specific and low single dose Etanercept combination therapy could be found.

To this end, we chose to measure by real time PCR (TaqMan) expression of two functional markers of Treg function: IL-10 and FOXP3. FOXP3 is a forkhead transcription factor whose expression is deemed crucial for Treg function. IL-10 is considered among the most important soluble mediators for regulatory T cell function. We also measured expression of TNFα, to evaluate if the different therapeutic regimens had a direct effect on the inflammatory response of effector CD4+CD25− cells.

**Figure 4:**
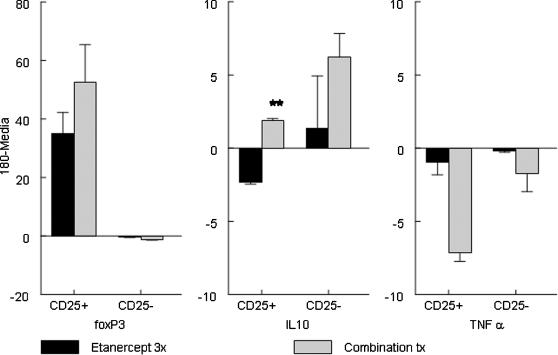
Combination Therapy led to an increase in FOXP3 and IL-10 gene transcription in CD4+CD25+ cells, whereas it also led to and increase in IL-10 transcription in CD4+CD25− cells. TNFα transcription was abolished by the combination therapy as well as by a full course of Etanercept treatment. Results are expressed as the induction index (marker/housekeeping gene GAPDH) of HSP60 peptide 180-188 stimulation subtracted by media alone as measured by Real Time Quantitative PCR. IL-10 production Combination therapy vs. Etanercept treatment p = 0.002. N = 2–4 per treatment.

CD4+CD25+ cells were studied, a category of Treg that appears functionally impaired in RA and whose efficiency might not be entirely restored by full dose anti-TNFα treatment [26]. CD4+CD25+ and CD4+CD25− MLN cells were isolated on day 23 after arthritis induction and cultured with HSP60 peptide 180-188 or media. After 15 hours viable cells were harvested, stained for CD4 and CD25 and sorted by FACS. Subsequently mRNA was extracted from sorted CD4+CD25+ and CD4+CD25− cells and levels of FOXP3, IL-10 and TNFα measured by Real Time Quantitative PCR. Figure 4 shows the ratio of the induction index (cytokine/transcription factor divided by housekeeping gene GAPDH) of stimulation with HSP60 peptide 180-188 less the background value.

When FOXP3 expression was measured, a significant increase was found both in the combination and full dose anti-TNFα groups, underscoring likely effects of both therapeutic regimens on some Treg functions, in accordance with recent findings in Rheumatoid Arthritis patients[26]. Cytokine mediated Treg function however might reportedly not be affected by anti-TNFα therapy, and indeed, when IL-10 expression by CD4+CD25+ cells was measured there was a significant increase (p = 0.002) only in the combination therapy group. Interestingly, combination therapy and, to a lesser degree, full dose Etanercept, also induced immune deviation of CD4+CD25− effector cells, with higher production of IL-10 consistent with what shown in the FACS analysis. As expected, FOXP3 expression was not induced in CD4+CD25− cells and TNFα expression was abolished by the combination treatment as well as by full dose Etanercept.

These data provide evidence for enhancement of Treg function by epitope specific and low dose Etanercept combination therapy. Enhanced or restored function of Treg led to immune deviation in effector CD4 cells, with production of IL-10. These immunological changes correlated with the changes in the clinical picture induced by the treatment.

### Adoptive Transfer of MLN T cells obtained from animals treated with epitope specific/low dose Etanercept combination therapy was able to treat full blown autoimmune arthritis

The purpose of this experiment was to evaluate whether the effects of the combination therapy on T cells could induce clinical amelioration upon adoptive transfer into sick animals. We employed T cells from spleen, Inguinal Lymphnodes (ILN) and MLN after combination treatment with Etanercept and 180-188 on day 23 after the induction of arthritis, cultured them for 48 hours with conA and subsequently injected them i.v. into the tail vein of rats one week after arthritis induction with Mt.

**Figure 5:**
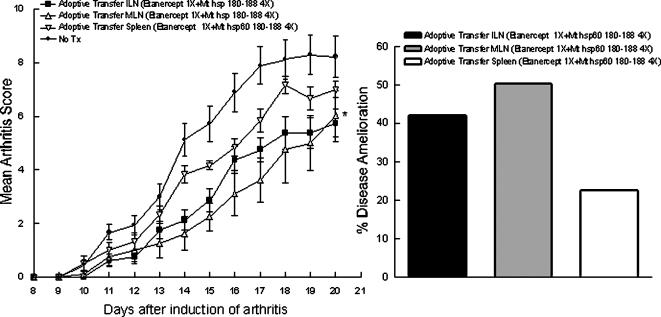
Adoptive Transfer of Mandibular Lymphnode (MLN) T cells from Combination Therapy treated animals led to significant reduction of Adjuvant Arthritis (AA) in diseased animals, measured as Mean Arthritis Score as well as percentage of Disease Amelioration. **A.** *Adoptive Transfer of T cells from Combination Therapy groups.* Adoptive Transfer Groups received 11×10^6^ Inguinal Lymphnode (ILN) cells, 13×10^6^ MLN cells, or 11×10^6^ spleen cells. Data represent Mean±SD. Disease induction and scoring was performed as described in the legend to Figure 1. **B.** *Percentage of Clinical Amelioration for each treatment group in AA rats*. The Area Under the Curve (AUC) of each individual treatment group was used to score the Clinical Amelioration (CA) of the distinct treatment groups. AUC was calculated using the curves originated by scoring the disease for the different treatment groups and plotted as percentage of CA with respect to the non-treated group. The non-treated group was considered as having an average percentage of disease = 100%. Formula is as follows: CA = 100 - %AUC.

Interestingly, only T cells derived from MLN of animals treated with the combination therapy were able to significantly reduce (p = 0.0305) clinical symptoms measured as mean arthritis score, when transferred into animals in which disease had been induced (Figure 5-A). Spleen cells from animals treated with combination therapy transferred to diseased animals failed to exert an efficient control of the disease process (Figure 5-B) as measured using percent of clinical amelioration. ILN were able to induce a good level of clinical amelioration (Figure 5-B), however differences with no treatment control group did not reach statistical significance (Figure 5-A).

Hence, epitope specific mucosal tolerization acts presumably on a population of T cells that resides in the lymphnodes draining the mucosa where the tolerization occurs (MLN) and to a lesser degree ILN. The effect of the treatment on T cells is lasting enough to allow efficient control of the disease process by adoptive transfer in animals in which AA was induced.

## Discussion

Recent years have witnessed a dramatic progress in our ability to understand mechanisms of autoimmune inflammation and to translate such understanding into novel therapeutic approaches. Particularly remarkable is the success of therapies aimed at interfering with the pro-inflammatory role played by certain cytokines, in particular TNFα. The broadening of clinical applications employing anti-TNFα therapy has led, however, to two interesting developments in clinical immunology, including: i) the recognition of significant generalized immune suppression in treated patients, with a sizable increase in onset or relapse of certain infectious diseases and neoplasias; ii) the need to understand in depth the effects of the treatment on the immune system.

In fact, the effects of anti-TNFα treatment on cytokine production and immunoregulation are still largely unknown and sometimes contradicting. Schotte et al. described the reduction of the number of PBMC producing the pro-inflammatory cytokines TNFα, IFNγ and IL-1 after Etanercept therapy, whereas the number of IL-10 producing PBMC remained the same, possibly indicating an immune suppression rather than active immunomodulation due to Etanercept[27]. Sieper and colleagues on the other hand investigated the effects of the treatment on the T cell population and postulated that neutralization of peripheral TNFα by Etanercept does not lead to a down regulation of the ability to produce TNFα or IFNγ by T cells, but rather to an up regulation, possibly due to a counter regulatory mechanism[28]. Ehrenstein et al.[26] found that Treg function in RA is impaired, and that treatment with Infliximab, a monoclonal antibody directed against TNFα, restored it only partially. Namely, Treg mechanisms based on cell-to-cell contact were restored by Infliximab treatment, while Treg mechanisms relying on soluble mediators such as IL-10 remained ineffective despite the treatment. A recent elegant study by Valencia et al. added important insight into the role of TNFα on T regulatory cells. They showed that CD4CD25^bright^ T regulatory cells constitutively express the TNF receptor II. An environment with high levels of circulating TNF led to up regulation of the TNF receptor II, which down regulated both the quantity as well as the quality of FOXP3+ T regulatory cells. Additionally they showed that CD4CD25^bright^ cells of patients with active RA expressed high levels of TNF receptor II, reduced levels of FOXP3 and were poor suppressors, which could be reversed by anti- TNFα treatment[13]. These studies, at times contradicting with respect to some mechanisms, underscored that short-term treatment with anti-TNFα may partially restore a more tolerogenic microenvironment, which could be instrumental for the induction of immune tolerance with epitope specific immune therapy.

Intervention on T cell mediated adaptive immunity would be, in theory, ideal, given the possibility of focusing the approach on one or more possible antigens involved in the disease process, thus sparing the patient generalized immune suppression. Progress is therefore needed in the area of modulation, rather than suppression, of T cells. The most important conceptual development may, however, be the fact that the search for the one inciting and still unidentified antigen should be replaced by approaches targeting mechanisms of control of self-reverberating T cell mediated inflammation. This would realistically shift the focus from etiology to pathogenesis based immune modulation.

A considerable body of evidence, to which we contributed[21], [36], [37], supports the concept that peptides derived from heat shock proteins (HSP) may play a role in amplification of autoimmune inflammation. As ubiquitous and bacterial derived products, HSP-derived peptides are in fact perceived as a “danger” signal and elicit a default pro-inflammatory physiologic response. Such response contributes in clearing a possible pathogen invasion but also induces, through cellular stress, increased availability of self-HSP derived peptides. These peptides are recognized by T cells with regulatory function. Such function is impaired in autoimmune arthritis[17], [18].

We have recently reported the results from a Phase I/IIa clinical trial in Rheumatoid Arthritis[25]. The objective of our clinical intervention was to restore natural mechanisms of immune modulation by exploiting the ability of the mucosal route in inducing tolerization to a HSP-derived peptide, which we previously described as part of the pro-inflammatory mechanisms of RA pathogenesis[21]. Interestingly, we were able to induce in treated patients immune deviation from pro-inflammatory to modulatory T cell responses, leading to significant reduction in TNFα and IFNγ production and increase in IL-10 and IL-4. These effects were mediated via restoration of function of CD4CD25^bright^ Treg, producing IL-10 and expressing FOXP3[25].

The study reported here addresses the questions on whether epitope specific and anti-cytokine therapy can be complementary, and if such synergy may be advantageous in order to exploit modulation of adaptive immunity while reducing generalized immune suppression, costs and side effects. In order to explore the concept, we chose AA, a T cell, HSP-dependent model of RA, which can be treated with full dose Etanercept. We have previously shown in AA that mucosal tolerization to the inciting peptide leads to immune deviation[31], [38].

Combination of epitope specific and anti-cytokine therapy induced full clinical control of AA, to a degree comparable to full dose Etanercept and significantly better than the other treatment regimens, including low dose Etanercept or epitope specific therapy alone. The comparable clinical efficacy achieved by combination treatment as well as full course Etanercept was obtained through distinctly different immune mechanisms in both effector T cells as well as regulatory T cells.

In effector CD4 cells, the combination therapy induced immune deviation while full dose Etanercept appeared to be eminently suppressive. Combination therapy led to an increased production of IL-10, which was not found in the other treatment regimens, including full dose Etanercept. Both treatments induced suppression of TNFα production and an increase of IL-4 production, which may indicate the presence of a T Helper 2 type tolerogenic mechanism complementing the main effects of the therapy.

Increasing evidence is, however, shifting the focus of modulation of adaptive immunity from effector to regulatory T cells[39]–[43]. Recent progress in Molecular Immunology has enabled the identification of phenotypical and functional characteristics for these T cells, such as co-expression of CD4, CD25 and CTLA-4, as well as production of certain regulatory cytokines. Several mechanisms of actions have been proposed for Treg, based either on release of cytokines with a tolerogenic function (eminently IL-10), or based on direct cell-to-cell contact by the use of receptors and pathways not yet fully elucidated[7], [39], [44]–[46].

When we sought to analyze the effects of combination therapy and full dose Etanercept therapy, it appeared evident that different functions of regulatory T cells were affected by the two treatments. Both treatments significantly increased the expression of the forkhead transcription factor FOXP3, a functional marker of Treg, which act eminently by cell-to-cell contact. A similar observation was described in human RA by Ehrenstein et al.[26]. However, CTLA-4 expression and IL-10 production were induced only by combination therapy regimens, and not by anti-TNFα treatment alone, again in agreement with what was found by Ehrenstein. Here we provide evidence that restoration of such function can be achieved by adding epitope specific immunotherapy to low dose Etanercept.

Undoubtedly, anti-TNFα therapy creates an environment in which epitope specific immunomodulation can be induced more efficiently. Further evidence for this concept was recently obtained by Bresson et al., who showed that combination of peptide therapy with anti CD3 enhanced the clinical improvement in experimental diabetes compared to anti-inflammatory therapy alone, also through the induction of CD25+FOXP3+ Tregs, as well as insulin specific Tregs producing IL-10 and TGFβ[47].

Recently, it was elegantly shown by Zanin-Zhorov et al, that HSP60 peptides enhance CD4+CD25+ regulatory T cell function via TLR2 signaling [48], thereby providing an additional explanation for the regulatory effects observed after combination therapy.

Further underscoring the profound immunological differences in mechanisms of action between full dose Etanercept and combination therapy, only cells derived from MLN of animals treated with combination therapy were able to control disease when transferred in sick animals. Cells with regulatory properties have been recently described as residing in the MLN [49].

This work lays the foundation for a swift translation of this novel immunotherapeutic concept in human Rheumatoid Arthritis. The implications, should this approach succeed, range from increasing the range of success and utilization of epitope specific immunotherapy, to reducing significantly the costs and undesirable effects of current first generation biologics.
